# The Influence of Buddhist Meditation Traditions on the Autonomic System and Attention

**DOI:** 10.1155/2015/731579

**Published:** 2015-06-04

**Authors:** Ido Amihai, Maria Kozhevnikov

**Affiliations:** ^1^Psychology Department, National University of Singapore, Block AS4, No. 02 07, 9 Arts Link, Singapore 117570; ^2^Martinos Center for Biomedical Imaging, MGH and Harvard Medical School, 25 Shattuck Street, Boston, MA 02115, USA

## Abstract

Cognitive and neuroscience research from the past several years has shed new light on the influences that meditative traditions have on the meditation practice. Here we review new evidence that shows that types of meditation that developed out of certain traditions such as Vajrayana and Hindu Tantric lead to heightened sympathetic activation and phasic alertness, while types of meditation from other traditions such as Theravada and Mahayana elicit heightened parasympathetic activity and tonic alertness. Such findings validate Buddhist scriptural descriptions of heightened arousal during Vajrayana practices and a calm and alert state of mind during Theravada and Mahayana types of meditation and demonstrate the importance of the cultural and philosophical context out of which the meditation practices develop.

## 1. Introduction

Providing scientific conceptualizations of meditation practices has been one of the major concerns of recent scientific studies of meditation [1–4]. One of the first scientific conceptualizations of meditation was proposed by Herbert Benson, who defined meditation as a technique that generates a “relaxation response” [5, page 56]. Benson conducted his studies on Transcendental Meditation (TM), where the meditator recites a mantra provided to him or her by the meditation instructor, as well as on Mindfulness meditation, which is a form of meditation that emphasizes the stabilization of attention by acknowledging discursive sensory events as momentary, and observing them without affective reaction or attachment. Benson showed that TM and Mindfulness meditation result in physiological changes indicative of a heightened activation of the parasympathetic nervous system and lowered sympathetic activity, such as decreased oxygen consumption and carbon dioxide elimination, lowering of heart and respiratory rates, and a marked decrease in arterial blood lactate concentration (e.g., [6, 7]), as well as psychological outcome measures that indicate relaxation (e.g., [8]). As these physiological and psychological results are characteristic responses that occur during relaxation, Benson termed the responses that occur during meditation as a relaxation response. Although Benson discovered the relaxation response by investigating TM and Mindfulness, he assumed that it applies to meditation in general and that it is useful to decontextualize different types of meditation from their religious and cultural basis: “to understand the psychophysiological aspects of meditation, it should first be conceptually denuded of its cultural and religious biases” [9, page 2]. Importantly, the attainment of a relaxation response during meditation has been confirmed by many subsequent studies and consistently reported in the scientific literature (e.g., [10–13]).

Based on Benson's approach, an evolutionary theory was proposed by Young and Taylor [14], where meditation was characterized as a “wakeful hypometabolic state of parasympathetic dominance” [14, page 149]. The hypometabolic state during meditation is a state of deep rest, which is similar to hibernation, but where the practitioner remains awake and vigilant [14, 15]. The state of being awake and vigilant was later termed “tonic alertness,” which indicates a state of optimal vigilance where attention is sustained for a prolonged period of time [16]. The state of “parasympathetic dominance” is generated by the increased activity of the parasympathetic branch of the autonomic nervous system, often referred to as the “rest and digest” system, which slows down the heart rate, lowers blood pressure, increases intestinal and gland activity, and relaxes sphincter muscles [17].

It is important to note that even though the characterization of meditation as a wakeful hypometabolic state is supported by empirical findings (e.g., [10–12]), the scientific studies that have led to this classification were conducted on very specific types of meditation. Specifically, most previous scientific studies have been conducted on TM and types of meditation from the Theravada and Mahayana traditions, such as Shamatha, Vipassana, or modern Mindfulness meditation. In particular, a large number of scientific studies were conducted on Shamatha or Vipassana [18, 19] that emphasize avoiding discursive thought by letting the practitioner concentrate on an object of meditation (Shamatha) or examine and generate insight out of his/her own mental activity (Vipassana) [20]. Also, many studies were conducted on Mindfulness meditation (e.g., [10, 12, 21]), which was developed by Jon Kabat-Zinn, who defined it as “mostly Vipassana practice… with a Zen attitude” (Kabat-Zinn email cited in [22], page 238), where elements from Theravada and Mahayana practices are taught alongside Vipassana meditation in order to create a secularized practice that would appeal to people who might not possess a genuine interest in Buddhist culture or philosophy [22]. (In accordance with this definition, Gilpin [22], who analyzed the influence of Buddhist traditions on Mindfulness, based on descriptions given by the developers of Mindfulness practices (Kabat-Zinn and John Teasdale), concluded that Mindfulness practice is mainly derived from Vipassana. Similar conclusions can be made by examining the practice of Mindfulness itself, which similarly to Vipassana, stresses avoiding discursive thought through nonjudgmental concentration on the content of one's momentary mental activity [23, pages 141-142]). A number of studies have also been conducted on Zen concentration meditation [24, 25], which similarly to Shamatha requires the meditator to continually focus on a single object of meditation [26, page 97].

Along with the large number of studies that confirmed that certain types of meditation can lead to a relaxation response, recent scientific evidence suggests that the generation of a relaxation response might not characterize meditative practices of other traditions. Specifically, meditative practices of the Vajrayana and Hindu Tantric traditions, which will be detailed in a subsequent section of the review, have been demonstrated to elicit a state of arousal and not relaxation [27–29]. In contrast to relaxation, arousal is a physiological and psychological state of being awake and reactive to stimuli. It is characterized by an increase in the activity of the sympathetic system, which is followed by the release of epinephrine and norepinephrine from the endocrine system [30–32] and results in the state of phasic alertness, a significant temporary boost in the capacity to respond to stimuli [16, 33, 34]. Moreover, while tonic alertness can happen concurrently with relaxation and a recent review of the literature showed that it can occur during Theravada and Mahayana styles of meditation [25], phasic alertness is a result of the activity of the sympathetic system and therefore inconsistent with the state of relaxation.

The goal of this review is to show that different types of Buddhist meditation techniques can lead to relaxation or arousal depending on the type of meditation that is practiced. We also plan to show that while some types of meditation generate increased tonic alertness, or vigilance, along with a state of relaxation and parasympathetic activation, other types of meditation lead to increased phasic alertness and generate an immediate and dramatic increase in cognitive performance on visual tasks, consistent with the state of arousal and sympathetic activation. In the following sections, we will first provide an overview of the autonomic system and the manner in which it underlies psychological and physiological states of relaxation and arousal and influences attentional processes that relate to phasic and tonic alertness. We will proceed with a review of scientific studies that demonstrate a relaxation response during Theravada and Mahayana types of meditation, which will be followed by a review of studies that demonstrate tonic alertness during these practices. Lastly, we will review studies that demonstrate an arousal response, as well as phasic alertness, during Vajrayana and Hindu Tantric practices.

## 2. Measuring Relaxation and Arousal, as well as Tonic and Phasic Alertness

Anatomically, the autonomic nervous system (ANS) consists of neurons from within both the central nervous systems (CNS) and the peripheral nervous system (PNS) and receives input from anatomic regions that integrate information from within the body and the external environment, such as the hypothalamus, nucleus of the solitary tract, reticular formation, amygdala, hippocampus, and olfactory cortex [35]. The functional role of the ANS is to monitor important visceral processes that operate largely below the level of conscious awareness, such as heart rate, breathing, and digestion [35].

The ANS is comprised of two major neurobiological subsystems that function both independently and in concert: the parasympathetic nervous system and the sympathetic nervous system. These two systems often elicit opposing actions, so that when one system enhances or activates a physiological response, the other system inhibits it. The sympathetic nervous system is often called the “fight or flight” system, which accelerates the heart rate, constricts blood vessels, and raises blood pressure in order to enable a quick and mobilizing response, often as a reaction to an immediate threat. On the other hand, the parasympathetic system is often referred to as the “rest and digest” system, which slows down the heart rate, lowers blood pressure, increases intestinal and gland activity, and relaxes sphincter muscles [17]. As mentioned, the increased capacity to respond to stimuli that is generated by the sympathetic system has been termed phasic alertness [16, 33, 34]. Therefore, phasic alertness requires the activation of the sympathetic system and cannot co-occur with a physiological state of parasympathetic dominance, which is the relaxation response. On the other hand, although tonic alertness is inconsistent with drowsiness and sleep, it can nevertheless occur concurrently with a moderate level of parasympathetic activation and, as described below, can also occur during relaxed states.

There are numerous experimental methods that have been used to demonstrate the activity of the sympathetic (arousal) and parasympathetic (relaxation) systems. One commonly used method is related to the heart rate variability (HRV), which is determined by the autonomic system [30] and is assessed through electrocardiographic measures (abbreviated as EKG or ECG). HRV can be measured through time domain methods or frequency domain methods. The EKG frequencies that are used as autonomic activity measures are high frequencies (HF), typically between 0.15 and 0.4 Hz [36–40], and the ratio between low and high frequencies (LF/HF) [30]. While some researchers proposed that low frequencies (LF), typically between 0.04 and 0.15 Hz [36–40], can be used as a marker of sympathetic modulation [41–44], others attribute LF to both sympathetic and parasympathetic influences [45, 46]. In contrast, increases in HF are universally attributed to the activity of the parasympathetic system [41, 45, 47]. Under ordinary circumstances, HF decreases indicate decreased parasympathetic and increased sympathetic activation [36, 48, 49], although it should be noted that in some extreme cases (e.g., physical exercise or extreme stress), increases in HF could accompany an increase in sympathetic response [36, 48]. A measure that is associated with HF is the respiratory sinus arrhythmia (RSA), which measures vagal modulations on heart rate and is correlated with the activity of the parasympathetic nervous system [50, 51]. Studies also investigated the heart rate pressure product—the product of the systolic blood pressure and the heart rate—and the double product—the product of the mean blood pressure and the heart rate—which index the myocardial oxygen consumption and load on the heart and are increased due to sympathetic system activation [52].

Another commonly used measure of autonomic activation is the galvanic skin response (GSR), where increases in GSR typically indicate increased sympathetic activation, and decreased GSR increased parasympathetic activity [53, 54]. Additionally, body temperature increases, or thermogenesis, are also determined by the sympathetic system [55, 56]. Also, several studies have used changes in blood pressure in order to investigate the state of the autonomic system. As described above, decreases in blood pressure (mean, systolic, and diastolic) are caused by the parasympathetic nervous system and blood pressure increases by the sympathetic system [17]. Moreover, relaxation responses were measured through self-report scales that are correlated either with negative emotions such as distress that indicate reduced relaxation (e.g., Global Severity Index (GSI), [57]) or positive emotional states that indicate increased well-being and relaxation (e.g., Positive States of Mind (PSOM), [58]).

Previous findings demonstrated that during arousal, there is a significant temporary boost in the capacity to respond to stimuli, indicative of phasic alertness, which is manifested by improved performance on a number of visual and memory tasks [59–61]. Hence, phasic alertness was determined to occur during states that lead to immediate improvements in performance on cognitive tasks [27, 29]. In order to determine the occurrence of tonic alertness, previous studies have used both neuroimaging and behavioral measures. In terms of behavioral measures, psychological studies have shown that performance on target detection tasks can improve with increased tonic alertness [62]. In terms of neuroimaging studies, neuroscience research has shown that tonic alertness is associated with neural activity in right hemisphere cortical areas and subcortical networks, particularly the dorsal anterior cingulate cortex (dACC), the dorsolateral prefrontal cortex (DLPFC), the anterior insula, the inferior parietal lobule, the thalamus, and the brain stem [34, 63, 64].

## 3. Meditation Traditions

There are three main Buddhist traditions that exist today: Theravada, Mahayana, and Vajrayana. Theravada has been the predominant religion of continental South Asia, and the* Tipitaka*, or* Pali Canon* [20], is its canonical text, which contains the earliest record of Buddha's teachings. The most prevalent meditative techniques of the Theravada tradition are Shamatha and Vipassana, which emphasize avoiding discursive thought by letting the practitioner concentrate on an object of meditation (Shamatha) or his/her own mental activity (Vipassana) [65]. In Buddhist scriptures, Shamatha practice relates to training in the concentration of attention, so that the practitioners are instructed to place undistracted attention on the object of meditation, while withdrawing their focus from other objects [65, 66]. Vipassana refers to insight into the true nature of reality, entailing an understanding of the impermanence of everything that exists, which is coupled with pacification (serenity) of the mind [67, pages 1287-1288 (IV.1410)].

The Mahayana tradition incorporated the canonical texts of the Theravada tradition, but also introduced a vast corpus of philosophical and devotional texts, with the most distinctive feature being the “great compassion,” an inherent component of enlightenment, which is manifested in bodhisattvas (enlightened beings). The Mahayana tradition is the largest major tradition of Buddhism and is prominent in North Asia. Mahayana Buddhists perform an assortment of different types of meditation practices, many of which are rooted in Shamatha and Vipassana practices, particularly types of meditation from the Tiantai Mahayana tradition [68]. Moreover, Zen concentration meditation is similar to Shamatha in the sense that it is also performed through prolonged concentration on an object of meditation, most commonly on the breath, in order to experience one-pointedness of mind or Samadhi [26, pages 57, 97].

The third tradition is Vajrayana Buddhism, which is often called Tantric Buddhism, and is a central tradition of Tibetan Buddhism that adopted elements of Hindu Tantric methods and Mahayana Buddhism [69, 70]. Although a number of practices in Vajrayana Buddhism originated in Mahayana (e.g., training in compassion/Six Paramitas) and Theravada (e.g., renunciation, impermanence, elements of Shamatha and Vipassana), they are practiced and integrated in a Vajrayana context [71]. There are three streams of Vajrayana practice:* Generation, Completion with Sign, and Completion without Sign*. Generation or development practices are performed during the first stage of the meditation practice [72]. A central generation practice that will be discussed in this review is self-generation-as-Deity practice (Tibetan “Kyerim”; hereafter referred to as Deity meditation), which involves visualizing oneself as a particular Deity, and holding the focus of attention on the internally generated image surrounded by his or her entourage. The completion stages are divided into completion practices “without sign” that include Rig-pa meditation, a practice during which the meditator visualizes the dissolution of the Deity and its entourage into emptiness and aspires to achieve a state of awareness that is devoid of conceptualization [73, 74]. Completion “without sign” are advanced tantric practices called “the six Yogas of Naropa” [75], one of which is g-Tummo, which will be discussed in this review. The g-Tummo Vajrayana practice is also called “psychic heat,” since it is associated with intense sensations of bodily heat in the spine [76–78] and involves breathing and muscle exercises, as well as visualization techniques that enable the meditators to generate and maintain mental images of flames at specific bodily locations that are accompanied by an intense sensation of heat in the spinal area.

Vajrayana practices are related to Hindu Tantric practices which were developed within ancient Hinduism and described in the Yoga-Sutras [79]. Hindu Tantric practices also distinguish different stages of practice. In the Dharana stage, the meditation consists of concentrative techniques of absorption in a single object of meditation, often in objects with religious significance such as deities [80, Chapter 8, page 2]. The practice of Dharana precedes Dhyana, during which the meditator disengages from a single object of meditation to a complete absorption in meditation, which leads to a nonconceptual state of consciousness [81, 82]. Hindu and Buddhist Tantric practices share some commonalities in the sense that both practices place an emphasis on concentrative visualization techniques on religious objects or deities, both describe similar stages of practice, and both place an emphasis on achieving a nonconceptual state of consciousness.

While meditative techniques of all Buddhist teachings stress liberation from all conceptual delusions, the means of achieving it are quite different. Specifically, Buddhist texts state that Theravada styles of meditation, such as Shamatha, Vipassana, or Mindfulness, are techniques that emphasize “internally steadying” or stabilize the “unstable mind” and cultivate the state of quiescence and tranquility, through which the nature of the mind could be seen without obstruction [67, pages 1287-1288 (IV.410)] [83, pages 152-153 (I.165), 335 (II.290)]. Similarly, certain Mahayana practices such as Zen concentration meditation also place an emphasis on one pointed concentration as well as calmness [26, pages 57, 97]. Vajrayana scriptures, by contrast, emphasize the training “which is not exactly the same as keeping the mind still and quiet” [84, page 118] but rather aims at the realization of “self-existing wakefulness” or “an awake quality” of the mind, free from dualistic thoughts, which is “like a radiant flame of a candle which exists all by itself” [84, page 88]. Furthermore, Vajrayana teaching emphasizes that the preoccupation with “being too calm” blocks “the recognition of self-existing wakefulness,” and that in a Vajrayana context, “it is sometimes said that stillness is not absolutely necessary…” [84, pages 85-86]. Thus, from a Vajrayana perspective, the conceptualization of meditation as a relaxation response seems to be incongruent with Tibetan views of Vajrayana Tantric practices, which do not presuppose relaxation but contain descriptions that are more consistent with the generation of an arousal response.

## 4. Relaxation and Tonic Alertness during Meditation

As alluded to the above, Buddhist texts describe Theravada types of meditation as practices that promote not simply a relaxed state of mind, but a state of relaxation and alertness [23]. Moreover, certain Mahayana practices, notably Zen concentration meditation, place a special emphasis on calmness [26, page 57]. Indeed, empirical evidence that some meditation practices generate a relaxation response can already be found in Benson's studies from the 1970s and 1980s (e.g., [8, 85]). Since then, dozens of studies have demonstrated that certain types of meditation from the Theravada and Mahayana traditions elicit a relaxation response, even after a brief period of meditation (e.g., 20 minutes: [86, 87]). Most of these studies investigated Mindfulness meditation, although there have been studies on Zen concentration and Vipassana as well (e.g., [10–13]). Such findings have been replicated many times in the past, and it is now widely accepted that Mindfulness meditation can be reliably used to alleviate the effects of stress and depression and to increase relaxation. In fact, before the term “Mindfulness Based Stress Reduction (MBSR)” was introduced to describe interventions that implement Mindfulness in order to reduce stress, these interventions were explicitly called “Stress Reduction and Relaxation Programs” (e.g., [88]). Moreover, based on Buddhist scriptures that emphasize not only relaxation, but also warn against the hindrances of drowsiness and sleep during the practice [89, 90], Britton et al. [25] hypothesized that meditation would promote not only relaxation but also a state of tonic alertness. In line with this hypothesis, Britton et al. [25] reviewed over 20 studies that demonstrate that certain types of meditation, mostly from the Theravada and Mahayana traditions, can activate neural areas that are associated with tonic alertness. Their review incorporated mostly Mindfulness and Zen practices, but also a Vipassana and Shamatha meditation study, as well as studies on non-Tantric Tibetan practices that are different from Vajrayana (e.g., focusing on a single dot: Brefczynski-Lewis et al. [91]), and Loving-Kindness Meditation that is practiced in all Buddhist traditions and is not specific to Theravada, Mahayana or Vajrayana [65, 84]. Importantly, Britton et al. [25] reviewed studies that showed that Theravada and Mahayana types of meditation can activate the dACC, DLPFC, the anterior insula, the inferior parietal lobule, the thalamus, and the brain stem, which are areas that are implicated in tonic alertness [25, 63].

### 4.1. Relaxation Response during Theravada and Mahayana Meditation

Several studies have used physiological measures in order to demonstrate increased parasympathetic activation during Theravada and Mahayana types of meditation. An influential and highly cited study in this regard was conducted by Tang et al. [92], who compared two groups of participants without Mindfulness meditation experience who were randomly assigned to one of two experimental conditions: half the subjects were required to perform “integrative body-mind training (IBMT),” which incorporates aspects of Mindfulness training for 20 minutes during a 5-day period, and the other half underwent a form of relaxation training that involved squeezing and relaxing individual muscle groups. Tang et al. [92] measured HRV and GSR before, during, and after training in either meditation or relaxation and showed that the participants in the meditation group demonstrated significantly more parasympathetic activation and less sympathetic activation than the relaxation control. Specifically, the IBMT group demonstrated increases in HF and lower GSR relative to the relaxation control, leading the authors to conclude that the meditation group significantly increased the activity of the parasympathetic system and decreased the activity of the sympathetic system relative to the relaxation control. Similarly, Ditto et al. [93] recruited 32 experimental subjects without meditation experience who were randomly assigned to either a type of Mindfulness meditation that involved body scanning, a control condition during which subjects performed muscle relaxation exercises, or a waiting-list control group. The authors measured respiratory sinus arrhythmia (RSA) through a vagal tone monitor, which, as mentioned, is a measure of respiration that is synchronized to the HRV and generated by the parasympathetic nervous system. Ditto et al. [93] showed that RSA was increased for the participants who performed Mindfulness meditation relative to the control subjects, indicating that the parasympathetic system was activated during the meditation, and a relaxation response occurred. Similar findings were demonstrated by Krygier et al. [94], who observed increases in HF and decreases in LF/HF during Vipassana meditation relative to a rest baseline, for 36 participants who participated in a Vipassana retreat. Moreover, recent physiological measures that were obtained during Zen concentration meditation also demonstrated an increase in the activity of the parasympathetic system during this practice as well. For example, Wu and Lo [86] compared the EKG activity of a group of 10 experienced meditators (an average of 6 years of experience) who performed a type of meditation called “Zen Chakra,” where practitioners concentrate on a “Chakra”—a nonphysical “energy point”—located inside the third ventricle of the human brain, to the EKG activity of a group of 10 participants without meditation experience. Wu and Lo [86] measured the EKG activity during 2 sessions, a 10-minute rest condition for both meditators and nonmeditators and a 20-minute condition of either meditation (for the meditators group) or rest (for the control group), and showed that the meditation resulted in an HF increase as well as decreased LF/HF relative to the nonmeditation control group. These results were interpreted to indicate that Zen Chakra meditation “appears to push the sympathovagal balance to parasympathetic dominance” [86]. In another study, Takahashi et al. [87] measured the EKG activity of 20 participants who were taught to perform Su-soku meditation, which is a Zen concentration practice that is performed by silently counting one's breaths, so that one inhales naturally and exhales when the numbers are recited. If other thoughts occur during the meditation, the participant is instructed to let them pass, and direct his or her attention back to the counting. As a control condition, participants were trained to breath at a rate of 0.25 Hz in tempo with the sound of a metronome, in order to account for the respiratory frequency influences on HRV. Takahashi et al. [87] measured the EKG activity during both the meditation and control condition and observed an increase in HF as well as a decrease in LF/HF, in the meditation relative to the control condition, which was interpreted to mean that the parasympathetic system was more active during the meditation relative to the control.

In addition, a multitude of studies have demonstrated that Mindfulness meditation can elicit a relaxation response using self-reports that are correlated with relaxation, and clinical stress reduction programs that implement Mindfulness meditation have been shown to reduce stress and increase relaxation even after 4 to 5 weeks of training [95, 96]. In one such study, Jain et al. [96] introduced a clinical intervention program that incorporated 5 sessions of Mindfulness meditation that was conducted in 4 sessions of 1.5 hours and an additional 6-hour session, over a period of 4 weeks. The participants who performed meditation were compared to participants in a “somatic relaxation (SR)” intervention that was performed over an identical time period and number of sessions, where they performed muscle relaxation techniques and breathing and imagery exercises, as well as to a waiting list control group. They found that the participants' ratings of psychological well-being significantly increased following the meditation intervention in comparison to SR, indicating an enhancement in positive emotional states and decreased stress. Similarly, in Mackenzie et al. [97], 30 participants were randomly assigned to a short 4-week Mindfulness meditation intervention or a waiting list control group, and they were instructed to practice meditation for at least 10 minutes a day, 5 days a week. The intervention and control participants completed a battery of questionnaires before and immediately after the 4-week training program that assesses the emotional and overall well-being. Mackenzie et al. [97] found that reported well-being, life satisfaction, and relaxation increased for the intervention group but remained stable for the control group, demonstrating increased relaxation as a consequence of Mindfulness meditation. Similar findings have been consistently reported dozens of times in previous research where Mindfulness meditation was typically practiced for an 8-week period (e.g., [10–12]), and the ability of Mindfulness meditation to alleviate stress, induce relaxation, and regulate anxiety is now a widely accepted phenomenon in scientific research as well as clinical contexts (e.g., [12, 98–101]).

Furthermore, several Yoga practices that emphasize relaxation techniques (e.g., Yoga Nidra), as well as concentration on a single object of meditation, such as the breath for a prolonged period of time (and are therefore similar to Theravada and Mahayana forms of meditation) also lead to increased relaxation and a reduction in stress. Just as in the case of Theravada and Mahayana types of meditation, the ability of these types of Yoga practices to induce relaxation and relieve stress has been found in previous studies and extensively discussed in the scientific literature [102–104].

### 4.2. Tonic Alertness during Theravada and Mahayana Meditation

As reviewed in Britton et al. [25], neuroimaging studies have shown that the activity in brain regions related to tonic awareness can be enhanced during Theravada and Mahayana types of meditation. As mentioned, tonic alertness is associated with neural activity in the dACC, DLPFC, the anterior insula, the inferior parietal lobule, the thalamus, and the brain stem [34, 63, 64]. In a study that demonstrated tonic alertness in both Vipassana and Shamatha, Manna et al. [105] measured the fMRI BOLD signal in 8 Theravada monks with an average of over 15000 hours of Shamatha and Vipassana meditation experience as well as in 8 novices with 10 days of meditation experience. Both groups of participants performed 3 experimental blocks inside the fMRI scanner, each of which began with a 3-minute rest condition and was followed by 6 minutes of Shamatha meditation where they focused on their breathing, and 6 minutes of Vipassana meditation. They found stronger BOLD activation in brain areas related to tonic alertness in highly experienced meditators (monks) that performed both Shamatha and Vipassana, and in novices that performed Vipassana meditation. Specifically, Manna et al. [105] found that when monks performed Shamatha, the fMRI BOLD signal in the left and right dACC, DLPFC, and anterior insula was stronger than during the rest control condition. Moreover, for the monks, Vipassana meditation led to an increased signal in the DLPFC area. On the other hand, for the novice meditators, Shamatha meditation did not show an increased BOLD signal that is related to tonic alertness, possibly due to insufficient expertise with this practice, but Vipassana meditation led to increased activation in the left dACC, indicative of enhanced tonic alertness.

Allen et al. [106] recruited 61 participants without any meditation experience, who were randomly assigned to either a 6-week Mindfulness meditation course, or a 6-week “active-control” condition that involved group readings and discussions. This control condition was hypothesized to “cultivate absorption and thought related processes into an imaginary narrative, in contrast to the present-centered and open-monitoring aspect of mindfulness” [106]. Allen et al. [106] found increased DLPFC activation in the meditation group relative to the control and also found that the amount of meditation practice positively correlated with the level of dACC activation that occurred during the meditation. As reviewed by Britton et al. [25], similar findings of increased activation in neural areas related to tonic alertness have been observed in additional studies on Vipassana (e.g., [107, 108]), Mindfulness (e.g., [109]), and Zen meditation (e.g., [110, 111]).

Additional evidence that Theravada types of meditation lead to tonic alertness was obtained by several recent studies that have shown that Mindfulness (e.g., [112, 113]) and Shamatha [114] types of meditation can lead to a decrease in the activity of neural structures that are more active during unfocused activity than during attention demanding tasks [115–117]. These areas include the ventral medial prefrontal cortex, posterior cingulate, inferior parietal lobule, and the dorsal medial prefrontal cortex, which have been collectively termed the brain's “default network” [118].

Furthermore, recent studies have investigated the influence of meditation on tonic alertness using behavioural tasks that are specifically designed to measure alerting attention. In Jha et al. [119], the authors utilized the “attentional network test” (ANT), where in each trial, participants are presented with an arrow target that is surrounded by distractor arrows that point in either the same direction (congruent flankers) or a different direction (incongruent flankers) as the target. Moreover, the target is preceded by one of three cue conditions: a valid cue, a spatially neutral central cue, a spatially neutral double cue, or a no cue condition. Importantly, the efficiency of alerting attentional processes is assessed by the ANT as the reaction-time (RT) difference between the double cue and the no cue conditions. Jha et al. [119] assessed changes in tonic alertness in 3 groups of participants: one group participated in an intensive Shamatha retreat for 30 minutes a day for 8 weeks. The second group practiced Shamatha for 30 minutes a day for 8 weeks, and a third group did not undergo any meditation training. They compared tonic alertness at the beginning of the study (Time-point 1) and following the intervention (retreat or meditation program: Time-point 2). Jha et al. [119] showed that although the efficiency of alerting attention did not differ at Time-point 1 between the three groups of participants, at Time-point 2, the alerting attentional mechanisms of participants who attended the retreat became significantly more efficient (the RT difference between the no cue and double cue conditions was reduced relative to Time-point 1), and the magnitude of the improvement in alerting attention was correlated with the meditation experience of the retreat participants. Moreover, the increase in alerting efficiency was driven by a decrease in RT to no cue trials and suggests that the meditators became more vigilant and their tonic alertness increased.

In MacLean et al. [120], the authors measured the influences of Shamatha meditation on tonic alertness using a sustained-attention task, where single lines were presented at the centre of the screen while participants fixated on a small yellow dot. The lines could be either long (90% of the time) or short (10% of the time), and the participants were required to respond to the short lines by pressing a response button. They recruited 29 participants [120] with an average experience of 2668 hours of meditation, who participated in a 3-month Shamatha meditation retreat that consisted of 5 hours of meditation training per day. The sustained attention test was administered before, at the midpoint, and after the meditation retreat. The nonparametric index of perceptual sensitivity, *A*′, was calculated from the hit rates and false alarm rates for each of the 8 120-trial experimental blocks of the sustained attention task. Since the decline in *A*′ was largest during the first 4 blocks, MacLean et al. [120] defined improvements in tonic alertness as positive changes in the slope of *A*′ during the first four blocks. MacLean et al. [120] showed that the slope of *A*′ was more positive at the midpoint and after the meditation retreat, in comparison to the pre-retreat baseline, demonstrating that Shamatha meditation reduced the decrement in *A*′ that occurs during the sustained attention task and therefore increased tonic alertness. The authors interpreted their results to indicate that meditation training can “decrease resource demands and thus improve vigilance (tonic alertness)” [120].

Hence, the findings of studies that investigated tonic alertness through behavioural measures complement neuroimaging studies, and together they demonstrate that certain Theravada and Mahayana types of meditation can increase the activity in neural areas related to tonic alertness, which in turn leads to improvements on tasks that require tonic alertness. The recent findings of tonic alertness during Theravada and Mahayana meditation are important because they complement the picture portrayed by Buddhist scriptures, which describe meditation as a practice that generates both a relaxed and an alert state of mind [23, 26]. Moreover, our review is consistent with previous findings and reviews that consistently demonstrated a relaxation response during Theravada and Mahayana types of meditation [10–13].

## 5. Arousal and Phasic Alertness in Vajrayana Practices

As opposed to Theravada meditative practices, Vajrayana practice does not cultivate relaxation but an arousal response. Vajrayana Buddhist scriptures emphasize the realization of “self-existing wakefulness” or “an awake quality” of the mind and warn against excessive tranquility [84], in contrast to Theravada scriptures that emphasize quiescence and tranquility [67, pages 1287-1288 (IV.410)], as well as Mahayana meditation instructions that also emphasize calmness (e.g., [26], page 57). Furthermore, empirical evidence also suggests that arousal is generated during specific meditative practices. For instance, the generation of arousal during meditation has been observed in Hindu Tantric practices (e.g., [121, 122]) as well as in several Vajrayana practices (e.g., [27, 28]), although overall there have been far fewer studies on Vajrayana and Hindu Tantric practices than on Theravada and Mahayana.

Based on Theravada Buddhist scriptures that emphasize calmness and relaxation and Vajrayana scriptures that emphasize “wakefulness,” Amihai and Kozhevnikov [27] hypothesized that Deity and Rig-pa practices of the Vajrayana tradition would generate cognitive and physiological responses of arousal, while Vipassana and Shamatha practices of the Theravada tradition would generate a relaxation response. In order to investigate the autonomic activation that is generated during these types of practices, Amihai and Kozhevnikov [27] compared the EKG activity of experienced Theravada and Vajrayana meditators (with 8 and 7.4 years of meditation experience, resp.) as they practiced meditation. The Theravada types of meditation that were investigated were Vipassana meditation and Kasina meditation, which is a visualization type of Shamatha meditation in which the meditator focuses his or her attention on “Kasina objects” that are described in the Pali Tipitaka and are typically colored disks. The Vajrayana practices studied were Deity and Rig-pa practices. In this study, the participants performed a 10-minute rest condition that was followed by Shamatha and Vipassana meditation (15 minutes each) for the Theravada meditators, and Deity and Rig-pa meditation (15 minutes each) for the Vajrayana meditators. Moreover, the participants' EKG activity was monitored throughout the experiment. Amihai and Kozhevnikov [27] showed that Theravada types of meditation elicited increased HF (Vipassana) and decreased LF/HF (both Vipassana and Kasina) relative to the rest control condition, which is consistent with a relaxation response. On the other hand, Vajrayana practices produced increased arousal, as indexed by decreased HF (both Deity and Rig-pa) during the meditation relative to the control condition. Moreover, Kozhevnikov et al. [29] and Amihai and Kozhevnikov [27] presented experienced Theravada (an average of 8–12.3 years of experience) and Vajrayana meditators (an average of 7.4–13 years of experience) with 2 visual tests (the Mental Rotation Test (MRT) and Visual Memory Test (VMT: [123]), see [29] for details), which were performed before and immediately following a 20-minute session of Theravada meditation (Vipassana and Kasina: Amihai and Kozhevnikov [27]) or Vajrayana practices (Deity: Kozhevnikov et al. [29] and Rig-pa: Amihai and Kozhevnikov [27]). As mentioned, the dramatic improvements on visual tasks immediately following a stimulus or activity are indicative of enhanced phasic alertness. Hence, the authors hypothesized that improved performance on the visual tests immediately after the meditation practice would indicate that phasic alertness occurred during the meditation and that such an improvement would be observed following Vajrayana practices. In line with this hypothesis, the experimental results showed that only Vajrayana practices led to a large and immediate increase in performance on these tasks, while Theravada meditators did not demonstrate any improvement in their performance following the practice. Hence, these studies demonstrate that Vajrayana practices and not Theravada types of meditation, lead to an arousal response and phasic alertness.

Additional studies that demonstrated that Vajrayana practices can increase the activity of the sympathetic system and generate an arousal response were conducted on practitioners of g-Tummo meditation, which, as mentioned, is associated with intense sensations of bodily heat in the spine. Interestingly, Benson himself reported a phenomenon that was unclear to him at the time, that two of the three g-Tummo practitioners that participated in his study exhibited an activation of the sympathetic system as evidenced by increased metabolism and oxygen consumption [124], which is consistent with arousal and not a relaxation response. Moreover, in Kozhevnikov et al. [28], it was demonstrated experimentally that g-Tummo meditation can indeed raise the temperature of the body, which indicates a sympathetic response. Nonshivering generation of body heat—thermogenesis—is mediated by the sympathetic nervous system [55, 56]. In humans, thermogenesis is caused mainly by brown adipose tissue, which shunts the energy obtained from the oxidation of free fatty acids into heat, which is then distributed throughout the body via the adipose tissue vasculature [56]. Importantly, brown adipose tissue activity in humans is stimulated by the sympathetic nervous system [55]. Specifically, an increased discharge from supraspinal sympathetic premotor pathways results in the sympathetic activation of brown adipose tissue, which leads to thermogenesis [56]. By attaching a small thermometer in the armpit of highly experienced g-Tummo meditators (6–32 years of experience), Kozhevnikov et al. [28] were able to demonstrate for the first time that g-Tummo meditators can increase not only their peripheral but, more importantly, core body temperature during the meditation, demonstrating that the activity of the sympathetic nervous system significantly increases as a consequence of this practice. Notably, the thermogenesis induced during g-Tummo was so substantial that it raised the body temperature of the meditators above the normal body temperature range and into the range of slight or moderate fever (up to 38.3°C), reflecting an enhanced arousal response due to sympathetic activation. It should be noted that increases in the peripheral body (meditators' fingers and toes) temperature during g-Tummo were also found in Benson et al. [125]; however, the authors did not attribute such changes to increased sympathetic activation. In contrast, Benson et al. [125] speculated that increased sympathetic activation during meditation is “unlikely,” as it would be inconsistent with the parasympathetic activation that they observed in Theravada and TM types of meditation.

In addition to Vajrayana practices, several studies conducted on Hindu Tantric meditators demonstrated increased arousal. In an early but influential and often cited study, Corby et al. [121] recorded the GSR and heart rate during a Dharana type of meditation called Ananda Marga, which incorporates a focus on breathing while repeating a two-syllable word and ignoring external stimuli. They recruited 30 experienced Ananda Marga meditators (an average of 2.1 years and 3.1 hours of practice a day) as well as 10 subjects without meditation experience that served as controls. The GSR and heart rates were recorded during 3 experimental conditions: (1) a relaxation control; (2) an Ananda Marga meditation preparation condition, which involves paying attention to the breath while ignoring external stimuli; (3) Ananda Marga meditation, where the subjects were told to ignore external stimuli, pay attention to their breathing, and silently repeat a two-syllable word in phase with their breathing. In the control group, the participants chose their own word, while meditators used a personal mantra that they received from their meditation instructor. The results of Corby et al. [121] demonstrated that a state of arousal occurred during Ananda Marga meditation: (1) skin conductance (GSR) increased from the relaxation baseline to the meditation condition, but only for the meditators group; (2) a small heart rate increase was observed during the meditation condition relative to the baseline for the meditators group. Both of these measures indicated that sympathetic activation occurred during the meditation, demonstrating an arousal response.

Additionally, Telles and Desiraju [122] measured heart rate and GSR during a different type of meditation which requires concentration on a light source while contemplating a universal force. They recruited 18 experienced meditators (with an average of 10.1 years of experience) and compared the physiological measures obtained during meditation to those obtained by the same subjects during a control condition, which was similar to the meditation condition, but where random thinking was allowed and effortful concentration was not required. The findings of Telles and Desiraju [122] demonstrated that although there were no significant changes in GSR, an increase in heart rate occurred during the meditation condition, but not during the nonmeditation condition, relative to the baseline heart rate obtained prior to each condition. Hence, the findings of this study are indicative of increased sympathetic activation and an arousal response during this type of meditation.

To conclude this section, as opposed to Theravada and Mahayana types of meditation that demonstrate enhanced relaxation, investigations of Vajrayana and certain Hindu Tantric practices demonstrated increased arousal. This once again emphasizes the importance of philosophical and cultural influences on meditation and demonstrates that the term “meditation” is in many ways too general when used as a unified descriptor of all Buddhist and Hindu contemplative practices. These findings show that the supposition that “to understand the psychophysiological influences of meditation, it should first be conceptually denuded of its cultural and religious biases [9, page 2]” is highly misleading. The diametrically opposed findings, of relaxation in Theravada and Mahayana practices, and arousal in Vajrayana and Hindu Tantric practices point to the opposite conclusion. Namely, in order to understand the psychophysiological aspects of meditation, one needs to carefully examine its cultural and religious sources.

## 6. Conclusions and Future Directions

The aim of this review was to examine the scientific studies of meditation while focusing on the unique influences that different types of meditative traditions have on the activation of the autonomic system and attentional mechanisms. We have presented evidence, summarized in Table 1, which shows that Theravada and Mahayana types of meditation lead to increased parasympathetic activation that underlies a relaxation response, while Vajrayana and certain Hindu Tantric practices elicit enhanced sympathetic activation that underlies a robust arousal response. In addition, we outlined the cultural and philosophical motivations that have influenced these meditative practices. While Theravada and Mahayana scriptures emphasize that the purpose of meditation is to cultivate tranquility along with mental stability, Vajrayana scriptures describe practices whose purpose is to elicit states of enhanced arousal.

It is important to stress that the influences of Vajrayana and Hindu Tantric practices on physiology and behavior are only beginning to receive their due attention from the scientific community, and the long-term impact of Tantric practices is still not well understood. Hence, while it has been demonstrated that Vajrayana and Hindu Tantric practices can lead to immediate physiological changes that are coupled with improved cognitive performance, future studies should investigate the long-term cognitive and physiological changes that occur as a consequence of such practices. As previously mentioned, in contrast to Vajrayana and Hindu Tantric practices, many scientific studies have been conducted on Theravada or Mahayana types of meditation, and there is evidence that they can lead to long-term improvements on attentional tasks (e.g., [25, 120, 126]). It is thus prudent that similar long-term studies would be conducted on Tantric practices.

Related to the above, another important research direction that has yet to be explored is the long-term influence of Vajrayana and Hindu Tantric meditation on stress and well-being. As mentioned, the finding that Theravada types of meditation produce relaxation has resulted in their incorporation into clinical practices as stress reduction techniques (e.g., [10, 21, 95, 96]). Conversely, the finding that Vajrayana and Hindu Tantric types of meditation produce arousal suggests that they could be more problematic for people who are under a high level of stress. However, it is also likely that as they gain additional meditation experience, Vajrayana and Hindu Tantric meditators develop unique strategies to help them cope with stressful situations that could arise during their meditation practice, for instance, by transforming their negative emotions into the positive emotional states of the visualized Deity. Such possibilities should be examined in future studies.

In addition, future studies should investigate the specific mechanisms that underlie ANS changes during meditation, in order to understand the precise factors that mediate the arousal and relaxation responses. One possible mediating factor could be respiration changes. In this context, previous studies on Hindu breathing exercises called Pranayama, which often serve as preparatory exercises for meditation [79, page 310], showed that specific breathing techniques can be used to activate the sympathetic or the parasympathetic nervous systems (e.g., [127]). A second factor that should be considered is the specific visualization or other content that is used for meditation. For instance, it is plausible that a visualized Tibetan Deity would lead to a high degree of arousal due to the emotional content that it holds in the eyes of a Tibetan meditator, while the inward and outward flow of the breath that is being attended to during Shamatha meditation would not evoke intense emotional reactions.

In conclusion, although the scientific examination of the nature of meditative practices is not new, it is only in recent years that cognitive scientists and neuroscientists have begun to investigate the influences of the specific cultural and philosophical contexts out of which meditation practices developed on the psychophysiological outcomes of the practices. Such investigations have led to experimental breakthroughs that corroborate ideas laid down in Buddhist scriptures about the purpose and effects of meditation. Specifically, the scriptural descriptions of heightened arousal during Vajrayana practices and a relaxed and alert state of mind during Theravada and Mahayana types of meditation received empirical support, as research from the past several years demonstrated that Vajrayana as well as certain Hindu Tantric practices lead to heightened arousal and phasic alertness, while Theravada and Mahayana types of meditation elicit a relaxation response coupled with enhanced tonic alertness.

## Figures and Tables

**Table 1:** Summary of meditation studies of autonomic activation and alerting attention.

Study	Meditation type	Findings
Tang et al. [92]	Mindfulness (IBMT)	Increased HF and decreased GSR

Ditto et al. [93]	Mindfulness	Increased RSA

Jain et al. [96]	Mindfulness	Increased perceived positive emotions and decreased perceived distress

Mackenzie et al. [97]	Mindfulness	Increased perceived well-being, life satisfaction, and relaxation

Wu and Lo [86]	Zen concentrative meditation	Increased HF and decreased LF/HF

Takahashi et al. [87]	Zen concentrative meditation	Increased HF and decreased LF/HF

Manna et al. [105]	Shamatha	Increased fMRI BOLD signal in the dorsal anterior cingulate cortex (dACC), dorsolateral prefrontal cortex (DLPFC), and anterior insula in meditation experts

Manna et al. [105]	Vipassana	Increased dACC activation in meditation novices

Allen et al. [106]	Mindfulness	Increased DLPFC and dACC activation

Jha et al. [119]	Shamatha	Increased alerting network efficiency

MacLean et al. [120]	Shamatha	Improvement in a sustained attention task

Amihai and Kozhevnikov [27]	Shamatha (Kasina visualization)	Decreased LF/HF

Amihai and Kozhevnikov [27]	Vipassana	Increased HF and decreased LF/HF

Amihai and Kozhevnikov [27]	Rig-pa (Vajrayana Tantric)	Decreased HF and increased performance in mental rotation (MRT) and visual memory (VMT)

Amihai and Kozhevnikov [27]	Deity (Vajrayana Tantric)	Decreased HF

Kozhevnikov et al. [29]	Deity (Vajrayana Tantric)	Increased MRT and VMT performance

Kozhevnikov et al. [28]	g-Tummo (Vajrayana Tantric)	Increased thermogenesis

Corby et al. [121]	Ananda Marga (Dharana)	Increased GSR and heart rate

Telles and Desiraju [122]	Hindu Tantric meditation	Increased heart rate

## References

[1] Bodhi B. (2011). What does mindfulness really mean? A canonical perspective. *Contemporary Buddhism*.

[2] Gethin R. (2011). On some definitions of mindfulness. *Contemporary Buddhism*.

[3] Grossman P., Van Dam N. T. (2011). Mindfulness, by any other name…: trials and tribulations of sati in western psychology and science. *Contemporary Buddhism: An Interdisciplinary Journal*.

[4] Josipovic Z. (2010). Duality and nonduality in meditation research. *Consciousness and Cognition*.

[5] Benson H., Proctor W. (2011). *Relaxation Revolution: The Science and Genetics of Mind Body Healing*.

[6] Benson H., Rosner B. A., Marzetta B. R., Klemchuk H. P. (1974). Decreased blood pressure in borderline hypertensive subjects who practiced meditation. *Journal of Chronic Diseases*.

[7] Wallace R. K., Benson H. (1972). The physiology of meditation. *Scientific American*.

[8] Kutz I., Leserman J., Dorrington C., Morrison C. H., Borysenko J. Z., Benson H. (1985). Meditation as an adjunct to psychotherapy. An outcome study. *Psychotherapy and Psychosomatics*.

[9] Kutz I., Borysenko J. Z., Benson H. (1985). Meditation and psychotherapy: a rationale for the integration of dynamic psychotherapy, the relaxation response, and mindfulness meditation. *The American Journal of Psychiatry*.

[10] Chiesa A., Serretti A. (2009). Mindfulness-based stress reduction for stress management in healthy people: a review and meta-analysis. *The Journal of Alternative and Complementary Medicine*.

[11] Chiesa A., Serretti A. (2010). A systematic review of neurobiological and clinical features of mindfulness meditations. *Psychological Medicine*.

[12] Zeidan F., Martucci K. T., Kraft R., McHaffie J. G., Coghill R. C. (2014). Neural correlates of mindfulness meditation-related anxiety relief. *Scandinavica*.

[13] Ivanovski B., Malhi G. S. (2007). The psychological and neurophysiological concomitants of mindfulness forms of meditation. *Acta Neuropsychiatrica*.

[14] Young J. D.-E., Taylor E. (1998). Meditation as a voluntary hypometabolic state of biological estivation. *News in Physiological Sciences*.

[15] Jevning R., Wallace R. K., Beidebach M. (1992). The physiology of meditation: a review. A wakeful hypometabolic integrated response. *Neuroscience & Biobehavioral Reviews*.

[16] Petersen S. E., Posner M. I. (2012). The attention system of the human brain: 20 years after. *Annual Review of Neuroscience*.

[17] McCorry L. K. (2007). Physiology of the autonomic nervous system. *American Journal of Pharmaceutical Education*.

[18] Morse D. R., Martin J. S., Furst M. L., Dubin L. L. (1977). A physiological and subjective evaluation of meditation, hypnosis, and relaxation. *Psychosomatic Medicine*.

[19] Lutz A., Zelazo P. D., Moscovitch M., Thompson E. (2007). Meditation and the neuroscience of consciousness: an introduction. *The Cambridge Handbook of Consciousness*.

[20] http://www.accesstoinsight.org/tipitaka/.

[21] Grossman P., Niemann L., Schmidt S., Walach H. (2004). Mindfulness-based stress reduction and health benefits: a meta-analysis. *Journal of Psychosomatic Research*.

[22] Gilpin R. (2008). The use of Theravāda Buddhist practices and perspectives in mindfulness-based cognitive therapy. *Contemporary Buddhism*.

[23] Gunaratana B. H. (2002). *Mindfulness in Plain English*.

[24] Cahn B. R., Polich J. (2006). Meditation states and traits: EEG, ERP, and neuroimaging studies. *Psychological Bulletin*.

[25] Britton W. B., Lindahl J. R., Cahn B. R., Davis J. H., Goldman R. E. (2014). Awakening is not a metaphor: the effects of Buddhist meditation practices on basic wakefulness. *Annals of the New York Academy of Sciences*.

[26] Buksbazen J. D. (2002). *Zen Meditation in Plain English*.

[27] Amihai I., Kozhevnikov M. (2014). Arousal vs. relaxation: a comparison of the neurophysiological and cognitive correlates of Vajrayana and Theravada meditative practices. *PLoS ONE*.

[28] Kozhevnikov M., Elliott J., Shephard J., Gramann K. (2013). Neurocognitive and somatic components of temperature increases during g-Tummo meditation: legend and reality. *PLoS ONE*.

[29] Kozhevnikov M., Louchakova O., Josipovic Z., Motes M. A. (2009). The enhancement of visuospatial processing efficiency through buddhist deity meditation. *Psychological Science*.

[30] Camm A. J., Malik M., Bigger J. T., Breithardt G., Cerutti S., Cohen R. J. (1996). Heart rate variability—standards of measurement, physiological interpretation, and clinical use. *Circulation*.

[31] Chess G. F., Tam R. M. K., Calaresu F. R. (1975). Influence of cardiac neural inputs on rhythmic variations of heart period in the cat. *American Journal of Physiology*.

[32] Levy M. N. (1971). Sympathetic-parasympathetic interactions in the heart. *Circulation Research*.

[33] Weinbach N., Henik A. (2011). Phasic alertness can modulate executive control by enhancing global processing of visual stimuli. *Cognition*.

[34] Sturm W., de Simone A., Krause B. J. (1999). Functional anatomy of intrinsic alertness: evidence for a fronto-parietal-thalamic-brainstem network in the right hemisphere. *Neuropsychologia*.

[35] Low P., Porter R. S., Kaplan J. L. (2013). Autonomic nervous system. *The Merck Manual*.

[36] Billman G. E. (2013). The LF/HF ratio does not accurately measure cardiac sympatho-vagal balance. *Frontiers in Physiology*.

[37] Berntson G. G., Bigger J. T., Eckberg D. L. (1997). Heart rate variability: origins methods, and interpretive caveats. *Psychophysiology*.

[38] Molgaard K., Hermansen K., Bjerregaard P. (1994). Spectral components of short-term RR interval variability in healthy subjects and effects of risk factors. *European Heart Journal*.

[39] Bigger J. T., Fleiss J. L., Rolnitzky L. M., Steinman R. C. (1992). Stability over time of heart period variability in patients with previous myocardial infarction and ventricular arrhythmias. *American Journal of Cardiology*.

[40] Stein K. M., Borer J. S., Hochreiter C. (1993). Prognostic value and physiological correlates of heart rate variability in chronic severe mitral regurgitation. *Circulation*.

[41] Malliani A., Pagani M., Lombardi F., Cerutti S. (1991). Cardiovascular neural regulation explored in the frequency domain. *Circulation*.

[42] Kamath M. V., Fallen E. L. (1993). Power spectral analysis of heart rate variability: a noninvasive signature of cardiac autonomic function. *Critical Reviews in Biomedical Engineering*.

[43] Rimoldi O., Pierini S., Ferrari A., Cerutti S., Pagani M., Malliani A. (1990). Analysis of short-term oscillations of R-R and arterial pressure in conscious dogs. *The American Journal of Physiology—Heart and Circulatory Physiology*.

[44] Montano N., Ruscone T. G., Porta A., Lombardi F., Pagani M., Malliani A. (1994). Power spectrum analysis of heart rate variability to assess the changes in sympathovagal balance during graded orthostatic tilt. *Circulation*.

[45] Akselrod S., Gordon D., Ubel F. A., Shannon D. C., Berger A. C., Cohen R. J. (1981). Power spectrum analysis of heart rate fluctuation: a quantitative probe of beat-to-beat cardiovascular control. *Science*.

[46] Appel M. L., Berger R. D., Saul J. P., Smith J. M., Cohen R. J. (1989). Beat to beat variability in cardiovascular variables: noise or music?. *Journal of the American College of Cardiology*.

[47] Pomeranz B., Macaulay R. J., Caudill M. A. (1985). Assessment of autonomic function in humans by heart rate spectral analysis. *The American Journal of Physiology*.

[48] Morady F., Kou W. H., Nelson S. D. (1988). Accentuated antagonism between beta-adrenergic and vagal effects on ventricular refractoriness in humans. *Circulation*.

[49] Eckberg D. L., Mohanty S. K., Raczkowska M. (1984). Trigeminal-baroreceptor reflex interactions modulate human cardiac vagal efferent activity. *Journal of Physiology*.

[50] Yasuma F., Hayano J.-I. (2004). Respiratory Sinus Arrhythmia: why does the heartbeat synchronize with respiration rhythm?. *Chest*.

[51] Katona P. G., Jih F. (1975). Respiratory sinus arrhythmia: noninvasive measure of parasympathetic cardiac control. *Journal of Applied Physiology*.

[52] Gobel F. L., Norstrom L. A., Nelson R. R., Jorgensen C. R., Wang Y. (1978). The rate-pressure product as an index of myocardial oxygen consumption during exercise in patients with angina pectoris. *Circulation*.

[53] Martini H. M., Bartholomew E. F. (2001). *Essentials of Anatomy and Physiology*.

[54] Gutrecht J. A. (1994). Sympathetic skin response. *Journal of Clinical Neurophysiology*.

[55] Nedergaard J., Bengtsson T., Cannon B. (2007). Unexpected evidence for active brown adipose tissue in adult humans. *The American Journal of Physiology—Endocrinology and Metabolism*.

[56] Morrison S. F., Blessing W. W., Llewellyn-Smith I. J., Verberne A. J. M. (2011). Central nervous system regulation of body temperature. *Central Regulation of Autonomic Functions*.

[57] Derogatis L. R., Melisaratos N. (1983). The brief symptom inventory: an introductory report. *Psychological Medicine*.

[58] Horowitz M., Adler N., Kegeles S. (1988). A scale for measuring the occurrence of positive states of mind: a preliminary report. *Psychosomatic Medicine*.

[59] Robbins T. W. (1997). Arousal systems and attentional processes. *Biological Psychology*.

[60] Robbins T. W. (2005). Chemistry of the mind: neurochemical modulation of prefrontal cortical function. *Journal of Comparative Neurology*.

[61] Hasegawa R. P., Blitz A. M., Geller N. L., Goldberg M. E. (2000). Neurons in monkey prefrontal cortex that track past or predict future performance. *Science*.

[62] Posner M. I. (2008). Measuring alertness. *Annals of the New York Academy of Sciences*.

[63] Sturm W., Willmes K. (2001). On the functional neuroanatomy of intrinsic and phasic alertness. *NeuroImage*.

[64] Sadaghiani S., Scheeringa R., Lehongre K., Morillon B., Giraud A. L., Kleinschmidt A. (2010). Intrinsic connectivity networks, alpha oscillations, and tonic alertness: a simultaneous electroencephalography/functional magnetic resonance imaging study. *The Journal of Neuroscience*.

[65] Buddhaghosa B. (2010). *Visuddhimagga: The Path of Purification*.

[66] Wallace A. (2006). *The Attention Revolution: Unlocking the Power of the Focused Mind*.

[67] Bhikkhu B. (2012). *The Numerical Discourses of the Buddha: A Translation of the Anguttara Nikaya*.

[68] Zhiyi S. (2009). *The Essentials of Buddhist Meditation*.

[69] Samuel G. (2008). *The Origins of Yoga and Tantra: Indic Religions to the Thirteenth Century*.

[70] Harper K. A., Brown R. L. (2002). *The Roots of Tantra*.

[71] Panchen N., Gyalpo P. W. (1999). *Perfect Conduct: Ascertaining the Three Vows*.

[72] Rinpoche S. (1990). *Dzogchen and Padmasambhava*.

[73] Wangyal T. (1993). *Wonders of the Natural Mind: The Essence of Dzogchen in the Native Bon Tradition*.

[74] Goleman D. (1996). *The Meditative Mind: The Varieties of Meditative Experience*.

[75] Tsong-Kha-Pa, Mullin G. C. (2005). *The Six Yogas of Naropa*.

[76] Evans-Wentz W. Y. (2002). *Tibetan Yoga and Secret Doctorines*.

[77] Mullin G. H. (1997). *Readings on Six Yogas of Naropa*.

[78] Mullin G. H. (1996). *Tsongkhapa's Six Yogas of Naropa*.

[79] Taimni I. K. (1961). *The Science of Yoga*.

[80] Kripalvanand S. (1977). *The Science of Meditation*.

[81] Feuerstein G. (1996). *The Philosophy of Classical Yoga*.

[82] Nash J. D., Newberg A. (2013). Toward a unifying taxonomy and definition for meditation. *Frontiers in Psychology*.

[83] Walshe M. (1995). *The Long Discourses of the Buddha: A Translation of the Digha Nikaya (Teachings of the Buddha)*.

[84] Rinpoche T. U. (1999). *As It Is*.

[85] Benson H. (1975). *The Relaxation Response*.

[86] Wu S. D., Lo P. C. (2008). Inward-attention meditation increases parasympathetic activity: a study based on heart rate variability. *Biomedical Research*.

[87] Takahashi T., Murata T., Hamada T. (2005). Changes in EEG and autonomic nervous activity during meditation and their association with personality traits. *International Journal of Psychophysiology*.

[88] Kabat-Zinn J. (1982). An outpatient program in behavioral medicine for chronic pain patients based on the practice of mindfulness meditation: theoretical considerations and preliminary results. *General Hospital Psychiatry*.

[89] Nanamoli B., Bhikkhu B. (1995). Sekha-patipada Sutta (the practice for one in training). *The Middle Length Discourses of the Buddha: A New Translation of the Majjhima*.

[90] Gyatso T. (2001). *Stages of Meditation*.

[91] Brefczynski-Lewis J. A., Lutz A., Schaefer H. S., Levinson D. B., Davidson R. J. (2007). Neural correlates of attentional expertise in long-term meditation practitioners. *Proceedings of the National Academy of Sciences of the United States of America*.

[92] Tang Y.-Y., Ma Y., Fan Y. (2009). Central and autonomic nervous system interaction is altered by short-term meditation. *Proceedings of the National Academy of Sciences of the United States of America*.

[93] Ditto B., Eclache M., Goldman N. (2006). Short-term autonomic and cardiovascular effects of mindfulness body scan meditation. *Annals of Behavioral Medicine*.

[94] Krygier J. R., Heathers J. A. J., Shahrestani S., Abbott M., Gross J. J., Kemp A. H. (2013). Mindfulness meditation, well-being, and heart rate variability: a preliminary investigation into the impact of intensive vipassana meditation. *International Journal of Psychophysiology*.

[95] Agee J. D., Danoff-Burg S., Grant C. A. (2009). Comparing brief stress management courses in a community sample: mindfulness skills and progressive muscle relaxation. *Explore*.

[96] Jain S., Shapiro S. L., Swanick S. (2007). A randomized controlled trial of mindfulness meditation versus relaxation training: effects on distress, positive states of mind, rumination, and distraction. *Annals of Behavioral Medicine*.

[97] Mackenzie C. S., Poulin P. A., Seidman-Carlson R. (2006). A brief mindfulness-based stress reduction intervention for nurses and nurse aides. *Applied Nursing Research*.

[98] Singleton O., Hölzel B. K., Vangel M., Brach N., Carmody J., Lazar S. W. (2014). Change in brainstem gray matter concentration following a mindfulness-based intervention is correlated with improvement in psychological well-being. *Frontiers in Human Neuroscience*.

[99] Ekman P., Davidson R. J., Ricard M., Wallace B. A. (2005). Buddhist and psychological perspectives on emotions and well-being. *Current Directions in Psychological Science*.

[100] Ekman P., Davidson R. J. (1994). *The Nature of Emotion: Fundamental Questions*.

[101] Baer R. A. (2003). Mindfulness training as a clinical intervention: a conceptual and empirical review. *Clinical Psychology: Science and Practice*.

[102] Streeter C. C., Gerbarg P. L., Saper R. B., Ciraulo D. A., Brown R. P. (2012). Effects of yoga on the autonomic nervous system, gamma-aminobutyric-acid, and allostasis in epilepsy, depression, and post-traumatic stress disorder. *Medical Hypotheses*.

[103] Balasubramaniam M., Telles S., Doraiswamy P. M. (2013). Yoga on our minds: a systematic review of yoga for neuropsychiatric disorders. *Frontiers in Psychiatry*.

[104] Telles S., Singh N. (2013). Science of the mind: ancient yoga texts and modern studies. *Psychiatric Clinics of North America*.

[105] Manna A., Raffone A., Perrucci M. G. (2010). Neural correlates of focused attention and cognitive monitoring in meditation. *Brain Research Bulletin*.

[106] Allen M., Dietz M., Blair K. S. (2012). Cognitive-affective neural plasticity following active-controlled mindfulness intervention. *The Journal of Neuroscience*.

[107] Gard T., Hölzel B. K., Sack A. T. (2012). Pain attenuation through mindfulness is associated with decreased cognitive control and increased sensory processing in the brain. *Cerebral Cortex*.

[108] Holzel B. K., Ott U., Gard T. (2008). Investigation of mindfulness meditation practitioners with voxel-based morphometry. *Social Cognitive and Affective Neuroscience*.

[109] Farb N. A. S., Anderson A. K., Mayberg H., Bean J., McKeon D., Segal Z. V. (2010). Minding one’s emotions: mindfulness training alters the neural expression of sadness. *Emotion*.

[110] Pagnoni G., Cekic M. (2007). Age effects on gray matter volume and attentional performance in Zen meditation. *Neurobiology of Aging*.

[111] Yu X., Fumoto M., Nakatani Y. (2011). Activation of the anterior prefrontal cortex and serotonergic system is associated with improvements in mood and EEG changes induced by Zen meditation practice in novices. *International Journal of Psychophysiology*.

[112] Farb N. A. S., Segal Z. V., Mayberg H. (2007). Attending to the present: mindfulness meditation reveals distinct neural modes of self-reference. *Social Cognitive and Affective Neuroscience*.

[113] Berkovich-Ohana A., Glicksohn J., Goldstein A. (2012). Mindfulness-induced changes in gamma band activity—implications for the default mode network, self-reference and attention. *Clinical Neurophysiology*.

[114] Brewer J. A., Worhunsky P. D., Gray J. R., Tang Y.-Y., Weber J., Kober H. (2011). Meditation experience is associated with differences in default mode network activity and connectivity. *Proceedings of the National Academy of Sciences of the United States of America*.

[115] Gusnard D. A., Raichle M. E. (2001). Searching for a baseline: functional imaging and the resting human brain. *Nature Reviews Neuroscience*.

[116] Raichle M. E., MacLeod A. M., Snyder A. Z., Powers W. J., Gusnard D. A., Shulman G. L. (2001). A default mode of brain function. *Proceedings of the National Academy of Sciences of the United States of America*.

[117] Raichle M. E., Snyder A. Z. (2007). A default mode of brain function: a brief history of an evolving idea. *NeuroImage*.

[118] Buckner R. L., Andrews-Hanna J. R., Schacter D. L. (2008). The brain's default network: anatomy, function, and relevance to disease. *Annals of the New York Academy of Sciences*.

[119] Jha A. P., Krompinger J., Baime M. J. (2007). Mindfulness training modifies subsystems of attention. *Cognitive, Affective and Behavioral Neuroscience*.

[120] MacLean K. A., Ferrer E., Aichele S. R. (2010). Intensive meditation training improves perceptual discrimination and sustained attention.. *Psychological Science*.

[121] Corby J. C., Roth W. T., Zarcone V. P., Kopell B. S. (1978). Psychophysiological correlates of the practice of Tantric Yoga meditation. *Archives of General Psychiatry*.

[122] Telles S., Desiraju T. (1993). Autonomic changes in Brahmakumaris Raja yoga meditation. *International Journal of Psychophysiology*.

[123] MMVirtual Design L.

[124] Benson H., Malhotra M. S., Goldman R. F., Jacobs G. D., Hopkins P. J. (1990). Three case reports of the metabolic and electroencephalographic changes during advanced buddhist meditation techniques. *Behavioral Medicine*.

[125] Benson H., Lehmann J. W., Malhotra M. S., Goldman R. F., Hopkins J., Epstein M. D. (1982). Body temperature changes during the practice of g Tum-mo yoga. *Nature*.

[126] Tang Y.-Y., Lu Q., Fan M., Yang Y., Posner M. I. (2012). Mechanisms of white matter changes induced by meditation. *Proceedings of the National Academy of Sciences of the United States of America*.

[127] Raghuraj P., Telles S. (2008). Immediate effect of specific nostril manipulating yoga breathing practices on autonomic and respiratory variables. *Applied Psychophysiology Biofeedback*.

